# Increasing resistance to bacterial leaf streak in rice by editing the promoter of susceptibility gene *OsSULRT3;* *6*

**DOI:** 10.1111/pbi.13602

**Published:** 2021-05-08

**Authors:** Xiameng Xu, Zhengyin Xu, Ziyang Li, Muhammad Zakria, Lifang Zou, Gongyou Chen

**Affiliations:** ^1^ School of Agriculture and Biology/State Key Laboratory of Microbial Metabolism Shanghai Jiao Tong University Shanghai China; ^2^ National Agricultural Research Center Crop Diseases Research Institute Islamabad Pakistan

**Keywords:** bacterial leaf streak, rice, *OsSULRT3;6*, gene editing

Rice (*Oryza sativa* L.) is an important cereal crop consumed by almost half the world population and is vital for global food security. Bacterial leaf streak (BLS), which is caused by *Xanthomonas oryzae* pv. *oryzicola* (*Xoc*), is a devastating rice disease in Asia, Africa and Australia (Ji *et al.,* 2014; Nino‐Liu *et al.,* 2006). Like other Xanthomonads, *Xoc* utilizes the type III secretion system (T3SS) to translocate effector proteins directly into host cells to suppress plant immunity (Nino‐Liu *et al.,* 2006; Yuan *et al.,* 2021). Transcription activator‐like effectors (TALEs) act as virulence or avirulence factors and function as eukaryotic transcription factors in plant cell nuclei, where they bind to effector‐binding elements (EBEs) of targeted plant gene promoters via a TALE‐encoded central repeat region (CRR). The CRR consists of highly conserved tandem repeats of 34‐amino acids; the hypervariable 12th and 13th residues in each repeat determine nucleotide binding specificity and are referred to as repeat‐variable diresidues (RVDs) (Boch *et al.,* 2009; Moscou and Bogdanove, 2009). Identification of TALE‐targeted genes in plants is used to facilitate plant disease resistance breeding programmes for *X. oryzae* pv. *oryzae* (*Xoo*), which is closely related to *Xoc* (Eom *et al.,* 2019; Ji *et al.,* 2016; Oliva *et al.,* 2019; Xu *et al.,* 2019).

Relatively few TALE‐targeted genes have been identified in the interaction of rice with *Xoc*. One example is *OsSULTR3;6*, which is up‐regulated by Tal2g in *Xoc* strain BLS256. *OsSULTR3;6* encodes a predicted sulphate transporter in rice and is considered a susceptibility (*S*) gene for BLS (Cernadas *et al.,* 2014). In the *Xoo*‐rice pathosystem, the disruption of TALE‐binding elements in three *S* genes confers broad‐spectrum resistance to *Xoo* (Oliva *et al.,* 2019; Xu *et al.,* 2019); therefore, we reasoned that a possible strategy for increasing rice resistance to *Xoc* might be the loss of susceptibility (RLS) genes. We speculated that rice would gain RLS if the EBE sequence of *OsSULTR3;6*, recognized by Tal2g, is edited by CRISPR/Cas9 technology (Zhou *et al.,* 2014). In the present study, CRISPR/Cas9 was to disrupt the Tal2g‐recognized EBE of *OsSULTR3;6* in rice cultivar IRBB10, which is susceptible to *Xoc*.

The TALE gene *tal5d* of *Xoc* strain RS105 was previously characterized (Ji *et al.,* 2014; Wilkins *et al.,* 2015). Tal5d contains 17.5 central repeat units that are nearly identical to Tal2g in *Xoc* strain BLS256; the obvious difference is that the 10th RVD of Tal5d is ND rather than HD in Tal2g (Figure 1a). We speculated that Tal5d might bind to the same EBE as Tal2g (EBE_Tal2g_) based on prediction of the two TALE‐binding sites (Figure 1a). Furthermore, no other variants of Tal2g and Tal5d were identified in the available sequences of *Xoc* (Ji *et al.,* 2014; Wilkins *et al.,* 2015).

**Figure 1:**
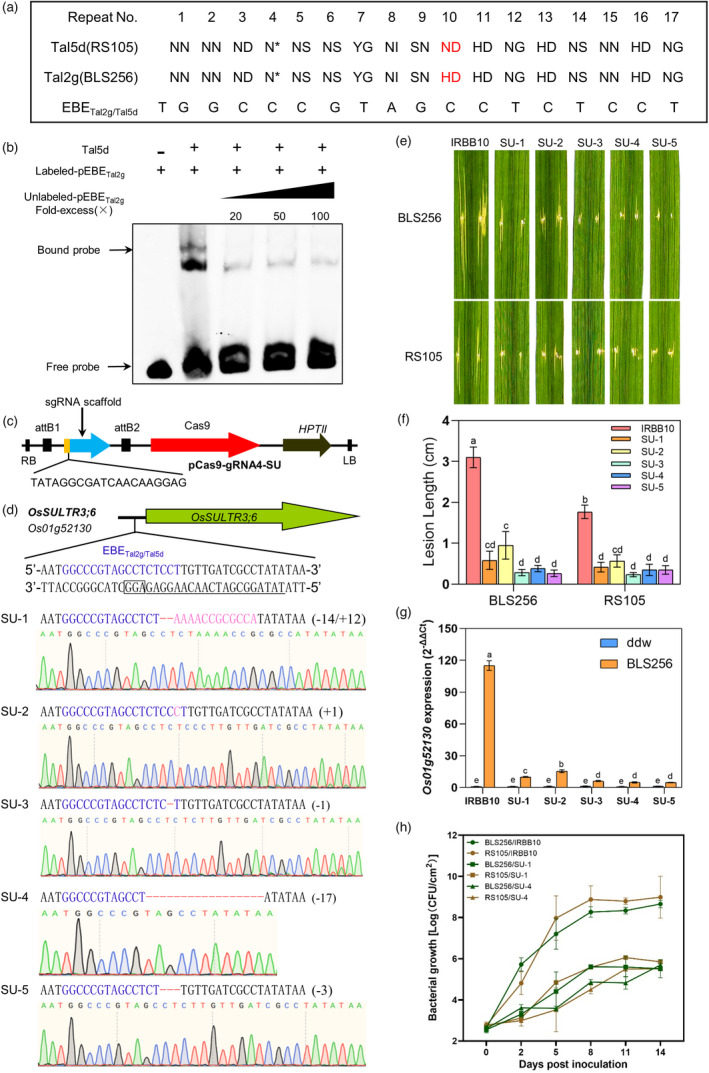
Generation of resistance to bacterial leaf streak in rice using CRISPR/Cas9. (a) RVD sequence alignment of Tal5d and Tal2g in RS105 and BLS256, respectively; the EBE_Tal2g/Tal5d_ consensus sequence is shown, and differences in the 10th RVD are indicated in red font. (b) *Xoc* Tal5d binds the EBE recognized by Tal2g (EBE_Tal2g_) in the *OsSULTR3;6* promoter. The uppermost sequence shows probe derived from *OsSULTR3;6* promoter, and the EBE_Tal2g_ is shown by blue letters. Unlabelled pEBE_Tal2g_ was used in competition assays. The presence and absence of DNA and proteins are indicated by (+) and (−), respectively. (c) Schematic diagram of pCas9‐gRNA4‐SU. RB, right border; LB, left border; and *HPTII*, hygromycin phosphotransferase II. The sequence shown below the map is for sgRNA. (d) Functional map of the *OsSULTR3;6* promoter in IRBB10 and analysis of edited rice lines. The uppermost diagram shows the *OsSULTR3;6* promoter with the EBE_Tal2g/Tal5d_ locus in blue font. Gene‐specific target sequences of sgRNA and PAM are underlined and boxed. The lower panel shows sequence analyses of homozygous lines SU‐1 to SU‐5. The red hyphen indicates a single nucleotide deletion. Pink letters indicate inserted nucleotides. (e) Disease phenotypes in rice IRBB10 and gene‐edited lines SU‐1 to SU‐5 (T_2_ generation) inoculated with *Xoc* strains BLS256 and RS105. (f) Disease lesion lengths on IRBB10, SU‐1, SU‐2, SU‐3, SU‐4 and SU‐5 at 14 dpi. Column height shows mean lesion length (cm), and error bars indicate the standard deviation of six replicates. Data are means ± SD of six biological replicates (Student's *t*‐test at *P* < 0.05). (g) *Os01g52130* expression in rice lines IRBB10, SU‐1 to SU‐5 infiltrated with *Xoc* strain BLS256. The expression of *Os01g52130* was evaluated by qRT‐PCR at 24 hpi. Data are means ± SD of three biological replicates (Student's *t*‐test at *P* < 0.05). (h) Bacterial growth of *Xoc* strains BLS256 and RS105 in leaves of rice line IRBB10, SU‐1 and SU‐4. Data points represent the mean ± SD from three replicates. All the experiments were repeated three times, and similar results were obtained.

To test this hypothesis, plasmid pET30a‐tal5d was constructed and used for purifying His‐Tal5d. Electromobility shift assays (EMSA) showed that the His‐Tal5d bound to the Cy5‐labelled pEBE_tal2g_ fragment, and binding was reduced by adding unlabelled pEBE_tal2g_ (Figure 1b). These results demonstrated that Tal5d binds the *OsSULTR3;6* promoter at the EBE_tal2g_ locus, which was then designated EBE_Tal2g/Tal5d_ (Figure 1a).

In order to disrupt Tal2g and Tal5d binding, we designed a sgRNA targeting the *OsSULTR3;6* promoter near EBE_Tal2g/Tal5d_ and constructed binary vector pCas9‐gRNA4‐SU (Zhou *et al.,* 2014) to edit the EBE_Tal2g/Tal5d_ sequence (Figure 1c,d). Five homozygous rice lines of IRBB10 (T_1_ generation) were obtained and named SU‐1 to SU‐5 (Figure 1d). PCR amplification and sequencing of the EBE_Tal2g/Tal5d_ region showed the following changes relative to the wild‐type EBE: SU‐1 contained a 14‐bp deletion and 12‐bp insertion; SU‐2 contained a 1‐bp insertion (cytosine); SU‐3 was missing a single nucleotide (thymine deletion); and SU‐4 and SU‐5 had 17‐ and 3‐bp deletions, respectively (Figure 1d).

The wild‐type IRBB10 and five edited lines (SU‐1 to SU‐5) were inoculated by pin‐pricking method (Pan *et al.,* 2018) with *Xoc* strains BLS256 and RS105, respectively, to investigate potential resistance. At 14 days post‐inoculation (dpi), the lesions induced by BLS256 and RS105 on the five edited rice lines (SU‐1 to SU‐5) were significantly smaller than those on IRBB10 (Figure 1e, 1f). It should be noted that SU‐2 line showed less resistance significantly than IRBB10, but the lesion length formed was obviously longer than those in SU‐1, SU‐3, SU‐4 and SU‐5 lines (Figure 1e,f). Sequence analysis showed that the single nucleotide insertion in SU‐2 occurred at the terminal nucleotide of the EBE (Figure 1d), suggesting that the modification may have less effect on TALE binding than the changes in the other four edited lines. The expression levels of *Os01g52130* in SU‐1 to SU‐5, infiltrated with BLS256, were significantly lower than those in IRBB10 (Figure 1g), suggesting that the edited EBE loci disrupt the activation of *Os01g52130* by Tal2g of BLS256 strain. For this, *Xoc* strains BLS256 and RS105, containing *tal2g* and *tal5d*, respectively, were then inoculated to IRBB10, SU‐1 and SU‐4 and bacterial growth was measured. Growth of *Xoc* BLS256 and RS105 was remarkably reduced in rice lines SU‐1 and SU‐4, respectively, as compared to the wild‐type IRBB10 (Figure 1h). These results indicated that the homozygous mutations in the EBE_Tal2g/Tal5d_ locus disarmed the recognition of Tal2g and Tal5d in rice nuclei, resulting in resistance to *Xoc* infection.

In summary, we first generated mutations in the EBE of the *OsSULTR3;6* promoter in IRBB10 and created new germplasm that exhibits resistance to *Xoc* strains containing virulence factors either Tal2g or Tal5d. Our findings show that genetic modification of the EBE_Tal2g/Tal5d_ sequence via CRISPR/Cas9 technology may be used to develop rice lines with broad‐spectrum resistance to bacterial leaf streak in rice by disrupting the EBEs of TALE‐matched *S* genes in rice.

## Conflict of interest

The authors declare no conflicts of interest.

## Author contributions

X.X. and G.C. designed the experiments. X.X., Z.X., Z.L. and M.Z. performed the experiments. X.X. and Z.X. wrote the manuscript. L.Z. and G.C. revised the manuscript.
